# Neoantigen Identification and Dendritic Cell-Based Vaccines for Lung Cancer Immunotherapy

**DOI:** 10.3390/vaccines12050498

**Published:** 2024-05-05

**Authors:** Komal Kumari, Amarnath Singh, Archana Chaudhary, Rakesh Kumar Singh, Asheesh Shanker, Vinay Kumar, Rizwanul Haque

**Affiliations:** 1 Department of Biotechnology, Central University of South Bihar, Gaya 824236, Bihar, India; komal@cusb.ac.in (K.K.); archana7893@gmail.com (A.C.); 2 Comprehensive Cancer Center, The Ohio State University, Columbus, OH 43210, USA; amarrnath.singh@osumc.edu; 3 Department of Biochemistry, Institute of Science, Banaras Hindu University, Varanasi 221005, Uttar Pradesh, India; rakesh_bc@bhu.ac.in; 4 Department of Bioinformatics, Central University of South Bihar, Gaya 824236, Bihar, India; 5 Heart and Vascular Institute, Pennsylvania State University, Hershey Medical Center, Hershey, PA 17033, USA; vinayktyagi07@gmail.com

**Keywords:** tumor-specific antigens, dendritic cells, neoantigens, next-generation sequencing, lung cancer

## Abstract

Immunotherapies can treat many cancers, including difficult-to-treat cases such as lung cancer. Due to its tolerability, long-lasting therapeutic responses, and efficacy in a wide spectrum of patients, immunotherapy can also help to treat lung cancer, which has few treatment choices. Tumor-specific antigens (TSAs) for cancer vaccinations and T-cell therapies are difficult to discover. Neoantigens (NeoAgs) from genetic mutations, irregular RNA splicing, protein changes, or viral genetic sequences in tumor cells provide a solution. NeoAgs, unlike TSAs, are non-self and can cause an immunological response. Next-generation sequencing (NGS) and bioinformatics can swiftly detect and forecast tumor-specific NeoAgs. Highly immunogenic NeoAgs provide personalized or generalized cancer immunotherapies. Dendritic cells (DCs), which originate and regulate T-cell responses, are widely studied potential immunotherapeutic therapies for lung cancer and other cancers. DC vaccines are stable, reliable, and safe in clinical trials. The purpose of this article is to evaluate the current status, limitations, and prospective clinical applications of DC vaccines, as well as the identification and selection of major histocompatibility complex (MHC) class I and II genes for NeoAgs. Our goal is to explain DC biology and activate DC manipulation to help researchers create extremely potent cancer vaccines for patients.

## 1. Introduction

Cancer ranks as one of the primary causes of both incidence and mortality in the current era. In 2020, the number of new cancer cases worldwide was 18.1 million, and approximately 9.5 million deaths were attributed to cancer. Projections indicate that these figures will rise to 29.5 million new cases and 16.4 million cancer-related deaths by 2040 [1]. According to GLOBOCAN 2020 estimates, lung cancer was the second greatest cause of both cancer-related incidence (11.4%; N = 2,206,771) and death (18.0%; N = 1,796,144) in 2020 [1]. Non-small cell lung cancer (NSCLC) and small cell lung cancer (SCLC) make up around 85% and 15%, respectively, of all lung cancer cases [2]. Early cancer stages are treated with surgery, radiation, and chemotherapy, whereas advanced cancer stages are treated with palliative care [3]. Although these therapeutic treatment procedures have had some accomplishments, they are not completely appropriate for malignancies that have spread to places where surgery is not possible, or chemotherapy/radiation is not permitted. Cancer immunotherapies (CIs) have emerged as a highly promising approach for combating cancer, potentially circumventing the limitations associated with conventional therapeutic modalities [4]. CIs comprise various treatment methods, such as oncolytic viruses, CAR T-cell therapies, antibody-based drugs, cancer vaccines, and other approaches [5]. Out of these treatments, cancer vaccines have emerged as a promising and highly effective approach for leveraging the immune system against cancer.

In the field of immunotherapy, the use of cancer vaccines that trigger T cells to treat growing tumors has gained more attention [6]. Cancer vaccines present a promising approach for stimulating a targeted and long-lasting immune response against tumor antigens (TAs). In malignant cells, TAs are primarily proteins that are overexpressed and are crucial for the development, growth, and spread of tumors [7,8]. There are two basic categories of TAs: tumor-associated antigens (TAAs) and tumor-specific antigens (TSAs). In TAAs, some non-mutated proteins are overexpressed or expressed in an abnormal way in cancer cells, while TSAs are formed from self-proteins that have undergone mutations and should not exist in healthy cells [9]. Other TSAs, which are a result of the genetic instability of cancer cells, may or may not be associated with the growth of tumors. They are traditionally known as “neoantigens” (Table 1).

Only one therapeutic cancer vaccine, a DC cancer vaccine (Sipuleucel-T), has been authorized for use in humans thus far. However, this vaccine has shown adverse effects in patients, prompting extensive research aimed at reducing these negative reactions. DNA, RNA, and peptide vaccines have been designed and developed to reduce negative adverse events but are still in the early stages of clinical trials, specifically in either phase I or II [10].

**Figure 1:**
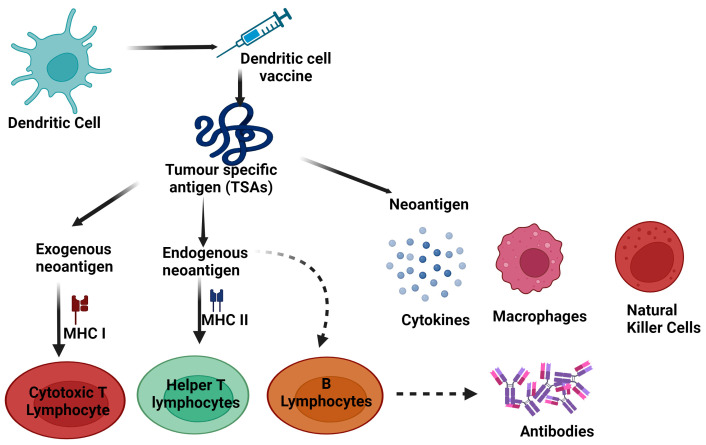
Schematic view of how tumor-specific antigens (neoantigens) initiate both innate and adaptive immune responses. Macrophages and natural killer (NK) cells participate in the innate response, directly targeting tumor cells. DCs present neoantigens via MHC class I molecules to activate cytotoxic T lymphocytes (CTLs), inducing tumor cell destruction. Simultaneously, DCs present antigens via MHC class II to T helper lymphocytes, prompting a cytokine release that amplifies CTL and B-cell activity. This intricate interplay unleashes a robust adaptive immune response, bolstered by various effectors like cytokines, macrophages, and NK cells. DC vaccines directly deliver neoantigen to DCs, facilitating antigen presentation to T cells and fostering a vigorous immune reaction against tumors. This orchestrated immune response, mediated by CTLs, T helper cells, B cells, and other effectors, underpins effective anti-tumor immunity, crucial for combating cancer progression(created with BioRender.com).

Cancer vaccines have the potential to stimulate the immune system, which in turn enhances the body’s defenses against cancer cells that express TAs. The effectiveness of therapeutic cancer vaccines primarily relies on the capacity of DCs to display TAs that contain CD4^+^ and CD8^+^ T-cell proteins. This is because DCs have the distinct capability to cross-present external antigens onto MHC I molecules, leading to the binding of cytotoxic T lymphocytes (CTLs) that specifically target the TAs [11]. Vaccine-based immunotherapy for cancer revolves around the utilization of DCs as a primary focus [12]. The present research considers the hurdles associated with immunotherapy and explores various vaccines produced, with a primary focus on targeting the antigen processing pathway of DCs to achieve maximum efficacy. Additionally, studies are also underway to examine the roles of adjuvants and alternate approaches to prevent immune escape mediated by tumors [13]. Cancer vaccines have the potential to stimulate the immune system, which in turn enhances the body’s defenses against cancer cells that express TAs. To accomplish this, cancer vaccines are designed to administer genes that encode TAs, thereby activating or boosting the immune system. In addition to activating the adaptive immune system, cancer vaccines can also stimulate the innate immune system by acting as potent “danger signals”. This, in turn, triggers multiple immune pathways in the cytosol of transfected cells; once these signals are detected by DCs, they initiate the uptake of antigens via various endocytic mechanisms [14]. Consequently, DCs can process foreign NeoAgs through two distinct pathways: presentation via MHC II to CD4^+^ T cells, or translocation into the cytosol for entry into the MHC I processing pathway, which enables “cross-presentation” to CD8^+^ T cells that are specialized for tumor recognition [15]. DCs can naturally acquire TAs in various forms, such as tumor lysates, dying or dead tumor cells, or immune complexes containing TAs, within the in vivo setting [16] (Figure 1).

The advancement of fundamental immunology has allowed for the development of more potent vaccine formulations, which has facilitated the progress of therapeutic vaccines designed for the treatment of infections into late-stage clinical trials with promising results [17]. However, the clinical efficacy of these therapies remains limited due to the high degree of variability in TAs and poor immune responses [18].

The primary aim of this review is to investigate and highlight the potential of DC-based vaccines against lung cancer. Additionally, we examined new methods to improve the presentation of antigens and address issues of low immunogenicity in DNA vaccines for lung cancer therapy. These strategies involve the use of novel delivery platforms, molecular adjuvants, and immunomodulatory factors as well as modulation of immune blockade. Furthermore, we emphasize the current clinical applications of DC vaccines in the treatment of lung cancer as well as explore the methods currently under investigation to overcome the limitations of cancer vaccines, in order to predict future developments in this area.

## 2. Immunotherapy in Lung Cancer

Immunotherapy has become a vital form of treatment that plays a crucial role in the care of patients with lung cancer. Notably, immunotherapy has shown encouraging results in treating both early and advanced stages of NSCLC and SCLC [19].

In recent years, there has been a renewed interest in the use of immunotherapy-based vaccines for the treatment of lung cancer. The aim of immunotherapy as a cancer vaccine is to facilitate tumor regression, eliminate residual malignancy, establish durable anti-tumor memory, and minimize non-specific or severe adverse effects [20]. Therefore, numerous delivery systems have been introduced for this purpose, including those utilizing DNA, RNA, viruses, and dendritic cells (DCs).

### 2.1. Genetic Vaccine

A genetic vaccine, also known as a gene-based vaccine, is a type of vaccine that utilizes nucleic acids, specifically DNA and RNA, to facilitate the production of antigen proteins within cells. DNA, RNA, viral, and DC-based vaccines are all examples of genetic vaccines.

#### 2.1.1. DNA Vaccines

DNA-based cancer vaccines have been proposed as a potentially effective strategy for activating the immune system to combat cancer. Previous clinical investigations utilizing DNA vaccines have demonstrated a favorable safety profile as well as the induction of a robust and targeted immune response. However, due to the immunosuppressive mechanisms of tumors, these vaccines have often exhibited only modest therapeutic effects in clinical trials [20]. To harness the full potential of DNA vaccination, an increasing number of preclinical and clinical studies are being conducted. The modulation of the immune system using DNA vaccination is a promising approach [21]. The potential of these substances lies in their ability to enhance the activation of T cells and stimulate the production of antibodies. Additionally, they offer the advantages of cost-effective manufacturing and long-term storage, while also exhibiting therapeutic properties [22], and their drawback is that the DNA molecules must pass through the nuclear membrane in order to be translated, despite the fact that they typically have low immunogenicity [23].

Various techniques are employed for the introduction of DNA into the nucleus, including popular methods such as electroporation and gene-gun vaccine delivery, which are commonly utilized for DNA vaccine delivery [21]. The electroporation method is employed to enhance the uptake of DNA by antigen presenting cells (APCs). This technique involves the application of brief electrical pulses, which cause temporary pores to form in the cell membrane. While electroporation has shown promise as a delivery approach for DNA vaccines, it is not without limitations. The injection process can cause significant pain and anxiety, making it unsuitable for large-scale vaccination programs [24]. A comparable method entails utilizing a gene gun to deliver DNA that is bundled with heavy metals (e.g., gold particles) to APCs. This technique has shown promising preclinical results, leading to phase I and II clinical trials for cervical cancer, and head and neck squamous cell carcinoma. Notably, this approach has resulted in a significant reduction in the amount of DNA required, by a factor of 100 to 1000 [24,25,26]. However, this approach has limitations in delivering vaccines to a large population, and thus alternative delivery systems are being explored.

#### 2.1.2. RNA Vaccines

The RNA-based vaccination platform provides several advantages, such as rapid production, the ability to encode numerous epitopes, and a reduced risk of integration into the host cell genome, thereby minimizing potential safety concerns [24]. However, these epitopes are present on TSAs. NeoAgs are a preferred antigenic target for vaccine development as they are exclusively expressed in tumor cells and not in healthy cells. Advanced formulations, such as liposomal delivery, have enabled intravenous administration, targeting APCs in all lymphoid compartments with the vaccine [27]. Due to the susceptibility of RNA to RNase degradation, various delivery technologies, including nanoparticles and liposomes, have been employed by many researchers to enhance transfection efficiency and prevent degradation [24,28,29]. Moreover, RNA has an advantage over DNA in that it only needs to be transported to the cytoplasm for protein translation, while DNA requires penetration into the nucleus for transcription.

One additional advantage of using RNA in vaccine development is its relatively simple and fast production process, which enables efficient scaling and cost-effective manufacturing within a shorter time frame [24]. Investigations are presently underway for personalized RNA vaccines that target NeoAgs in different types of tumors, such as NSCLC and melanoma (NCT03289962, NCT03815058, NCT04267237, NCT03313778, NCT03897881) [27].

Advancements in clinical trial design, frequency, and management have enabled the testing of vaccination strategies for cancer and infectious diseases. In response to the COVID-19 pandemic, national health officials have granted official approval to two RNA vaccines, namely Tozinameran from Pfizer-BioNTech and mRNA-1273 from Moderna [30]. An investigation was conducted to evaluate the efficacy of a NeoAg-encoding RNA vaccine in patients with melanoma through a clinical trial. Using a comparative exome analysis, NeoAgs were detected in the tumors of stage III and IV melanoma patients [24]. Furthermore, after translation into protein, RNA is processed by APCs into long peptides. These long peptides are then presented on the cell surface via MHC I or II molecules, which trigger and activate T cells. This mechanism of action is analogous to that of long peptide vaccines, as RNA vaccines also function in this manner. To minimize the likelihood of single antigen loss variants, RNA vaccines contain numerous NeoAg epitopes [24,31]. Despite having some limitations, a DC-based immunization strategy has been developed.

### 2.2. Virus-Based Vaccines/Recombinant Viral Vectors

In the context of a cancer vaccine initiative, several viruses have been utilized as vaccine vectors. Virus-based immunogens exploit the remarkable efficacy and adaptability of the host’s immune system in countering viral threats by synergistically engaging both innate and adaptive immune responses. This entails activating APCs through pattern-recognition receptors (PRRs) associated with viral pathogens [32]. Among the commonly employed viral vaccine vectors, poxviruses, adenoviruses, and alphaviruses stand out, with a preference for replication-deficient or attenuated variants to ensure safety. Viral vectors possess a disadvantage in that their efficacy can be impeded by the host’s antiviral immune response, which reduces or eliminates their efficacy, thereby limiting the efficacy of subsequent vaccinations. To address this challenge, various approaches are under investigation, including the heterologous prime-boost strategy. This strategy involves delivering a cancer antigen through one viral vector initially, then administrating a boost using a second viral vector, and then administrating a boost using a second viral vector or vector type (e.g., DNA plasmid) conveying the same TA [33]. To overcome these limitations, an alternative approach under investigation is RNA vaccination.

### 2.3. DC-Based Vaccines/Cell-Based Vaccines

DCs are a specialized type of APCs that possess the ability to efficiently capture both exogenous and endogenous antigens, and subsequently present them to CD8^+^ and CD4^+^ T cells via the MHC I and MHC II antigen presentation pathways, including the critical process of cross-presentation [34]. This cross-presentation is fundamental for generating and sustaining an effective and persistent immune response. DCs’ capacity to traverse the blood–brain barrier made them a potential therapeutic tool in the active area of cancer immunotherapy [35]. DCs exhibit their antigenic environment through various mechanisms such as phagocytosis, micropinocytosis, receptor-mediated endocytosis, and lectin-mediated endocytosis. Among these, immature DCs are more proficient in antigen processing. DCs undergo maturation upon encountering inflammatory mediators, such as bacterial lipopolysaccharide (LPS) or tumor necrosis factor alpha (TNF-α) [36]. Additionally, CD40 ligand interactions with CD40 induces DC maturation in helper T cells. While mature DCs have reduced antigen uptake and processing machinery, they possess increased expression of MHC molecules, costimulatory molecules (CD80 and CD86), and the chemokine receptor CCR7. In addition, mature DCs migrate to lymph nodes to stimulate T cells that are capable of recognizing the antigen, thus triggering an immune response against the presented antigen [37].

In recent years, there has been a growing popularity in the use of DCs that have been developed with TNF-α, the CD40 ligand, a monocyte-conditioned medium, or cytokine combinations [38,39]. Clinical trials are underway to test DC vaccinations against cancer. While several experiments involving DC cancer vaccines have been reported [40].

Recently, the use of DCs that have been developed with TNF-α, the CD40 ligand, a monocyte-conditioned medium, or cytokine combinations has become increasingly popular for anticancer DC vaccinations, which are currently undergoing clinical trials. Several studies have reported on DC cancer vaccine experiments, including those conducted by Steinman et al. (2001) [38] and Nestle et al. (2000) [39]. Although several DC cancer vaccine experiments have been discussed in the literature, the first DC vaccination research was reported by Hsu et al. (1996) [40]. However, this strategy still needs to overcome several technical challenges before it can be directly compared to other vaccine delivery systems. The use of therapeutic cancer vaccination with DCs shows potential in overcoming mechanisms used by cancers to evade the immune system [37]. Currently, various combinations of chemotherapies, checkpoint inhibitors, TLR agonists, tyrosine kinase inhibitors, and many more combined with DC-based vaccine delivery are being researched (e.g., NCT02688686, NCT03987867, NCT03360630, NCT01294306, NCT02766348). Although all of these strategies require some form of stimulus for the administration of the vaccine, several delivery system techniques have been developed, such as adjuvants, nanoparticles, and peptides.

## 3. Current Progress in Cancer Vaccine Delivery Systems

The delivery mechanism for cancer vaccines has undergone substantial progress, as it assists in enhancing vaccine immunogenicity and preventing degradation [41]. To achieve long-lasting effects, various adjuvant vaccines, nanoparticle vaccines, peptide-based vaccines, antigen delivery methods, adjuvants targeting toll-like receptors, and cytokines are required.

### 3.1. Antigen Delivery Systems

Studies conducted prior to clinical trials have shown that using incomplete Freund’s adjuvant (IFA) as a vaccine adjuvant can negatively impact the effector function, tumor localization, and survivability of vaccination-induced tumor specific T lymphocytes due to prolonged antigen presentation [42]. In acute pathogen infections, the primary wave of antigen typically dissipates within a week after priming T cell effectors, although smaller amounts of antigen can be stored in APCs for a longer period. It would be intriguing to explore whether this concept is widespread and whether it can enhance the efficacy and potency of peptide-based cancer vaccines [43].

### 3.2. Nanoparticles as Vaccine Delivery Systems

The delivery of drugs and antigens via nanoparticles is an intriguing approach. First, these particles can extend the in vivo half-life of encapsulated antigens and immunomodulators by shielding them from serum/tissue peptidases/proteases and other potentially damaging agents. Second, nanoparticles can be genetically modified to selectively target particular cell types or organs, including lymph nodes [44]. Incorporating drugs and antigens into nanoparticles has the potential to reduce off-target negative effects and lower the required dosage. Specifically, polylactic-co-glycolic acid (PLGA) particles encapsulating antigen (Ag) have shown to elicit a comparable T-cell response with a 1000-fold lower dose than free Ag [43,45].

Since the advent of nanotechnology, peptides coupled with nanoparticles have exhibited significant potential for treating diseases, especially cancer. The use of a synthetic substance (LFC131-DOX NPs) consisting of doxorubicin, PLGA nanoparticles, and a peptide (LFC131, a CXCR4 inhibitor) demonstrated a strong binding affinity towards human lung cancer cell line “A549” [46]. These findings suggest that LFC131-DOX NPs could be used as a drug delivery system for treating cancer. In a recent study, it was reported that nanoparticles loaded with docetaxel and attached to the TH10 peptide (TH10-DTX-NP) may be a promising therapeutic for cancer treatment in the rat lung metastasis model, specifically targeting vascular pericytes [47]. Similarly, chitosan microparticles containing bradykinin potentiating peptide (BPP) were investigated and found to increase vascular permeability in tumors, leading to enhanced drug accumulation and prolonged effects [48,49]. Furthermore, lipid nanoparticles (LNPs) have been shown to influence DC maturation based on the context, with nonadjuvanted LNPs promoting tolerogenic maturation and adjuvanted LNPs inducing immunogenic maturation, highlighting the adjuvant properties of the delivery system in shaping DC responses [50].

### 3.3. Immune Blockade

Immune checkpoint blockades use medications to block proteins that regulate the immune system. By inhibiting these proteins, the immune system can better identify and fight cancer cells. In cancer immunotherapy, the most common immune blockades are PD-1 inhibitors: Pembrolizumab (Keytruda), nivolumab (Opdivo) [51]; PD-L1 inhibitors: Atezolizumab (Tecentriq), durvalumab (Imfinzi) [52]; CTLA-4 inhibitors: Ipilimumab (Yervoy) [53]; TIM-3 inhibitors: LY3321367 [54]; LAG-3 inhibitors; TIGIT inhibitors: Tiragolumab, vibostolimab, etigilimab [55]; and VISTA inhibitors: JNJ-61610588 [56]. These medications boost immune responses against tumors by inhibiting the interaction between immune cells’ inhibitory checkpoint molecules and their ligands on tumor cells or other cells in the tumor microenvironment (TME). Immune blockades, ligands, and their mechanisms of action are listed in Table 2.

**Figure 2:**
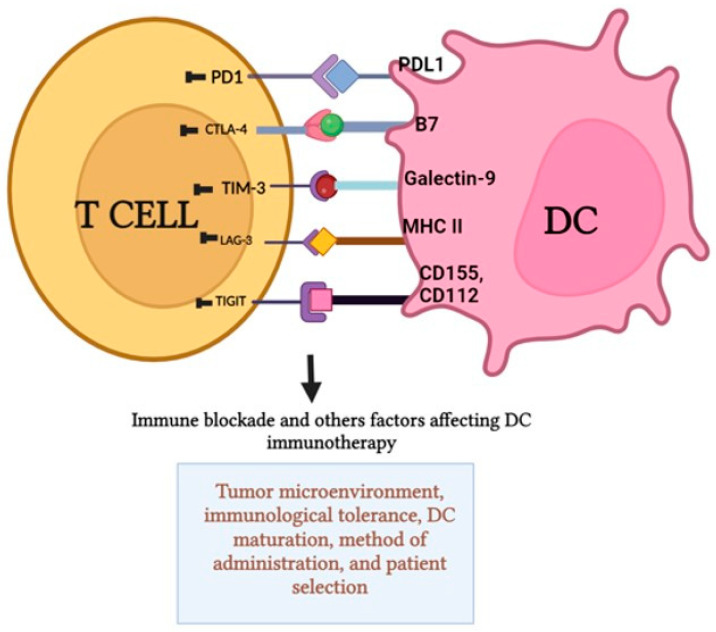
Blockades of immune checkpoints such as PD1, CTLA-4, TIM-3, LAG-3, and TIGIT have been shown to improve the effectiveness of DC therapy. However, excessive activation of these checkpoints can actually hinder the function of DCs and dampen the anti-tumor immune responses. Various factors, including immune blockades and other elements, have an impact on the efficacy of DC therapy. Additionally, the failure and suppression of DC therapy can be influenced by factors like the tumor microenvironment (TME), immune tolerance, DC maturation, route of administration, and patient selection.

These blockades and other factors enhance the efficacy of DC therapy. On the other hand, excessive activation of immune checkpoints can lead to the suppression of DC function and the inhibition of anti-tumor immune responses. Different factors like immune blockades and other factors affect the DC therapy. Other factors that can contribute to the failure and suppression of DC therapy include the tumor microenvironment (TME), immune tolerance, DC maturation, route of administration, and patient selection (Figure 2).

## 4. Ongoing Challenges in the Development of Lung Cancer Immunotherapy and Therapeutic Cancer Vaccines

Multiple NeoAg-specific immunotherapeutic clinical trials are currently underway worldwide to evaluate the effectiveness of various existing and novel immunotherapeutic agents for managing lung cancer. Table 3 highlights several recent trials that have been conducted on NSCLC and SCLC patients. However, creating a NeoAg-specific immunotherapeutic cancer vaccine poses specific challenges [62]. The limitation in NeoAg prediction accuracy poses a significant challenge for the extensive implementation of tailored immunotherapies.

This is due to the inadequate identification of tumor-specific cancer NeoAgs caused by the variations in mutational loads and divergent NeoAg presentation patterns observed across different tumor types [77]. The ability of cancers to avoid immune system identification is one of the obstacles to employing NeoAg-based immunotherapies [78]. The absence of NeoAg modifications to the way antigen peptides are delivered and the development of immunosuppressive milieus within the tumor are just a few of the mechanisms by which this evasion might take place [79]. In addition, tumors have the ability to modulate protein turnover, which can affect NeoAg presentation. Mutated proteins are prone to misfolding and quick degradation by the proteasome, which can result in an increase in the presentation of antigens [80]. Elimination of the entire sub clonal cell population through CD8+ T cell-mediated eradication can lead to the elimination of NeoAgs. Patients’ T cells recognize several deletion mutations, and tumors with substantial immune cell infiltration are less likely to generate genes encoding NeoAgs, suggesting that the immune system may preferentially eliminate NeoAg-expressing tumor subclones [81,82].

Tumors can experience mutations that impact not only the production of NeoAgs but also the diversity of the HLA molecules and the stability of the MHC complexes. These alterations can lead to impaired processing and presentation of NeoAgs, resulting in a reduction in T-cell recognition and impaired tumor elimination [83,84]. The immunosuppressive TME represents an additional challenge, which includes the inhibition of immune checkpoints, the immunosuppressive effects of various TME cells, and the release of ions or proteins from necrotic tumor cells. All of these factors can impede NeoAg recognition and T-cell activation [85]. Ultimately, a reliable immune surveillance system will be necessary to evaluate NeoAg-based immunotherapy [78].

## 5. Common Cancer Antigens in Lung Cancer

### 5.1. Personalized Vaccines Targeting Neoantigens

Extensive whole exome sequencing studies have shown that NSCLC has the highest mutational load among all types of cancers, with hundreds of non-synonymous somatic mutations present in each patient [86,87]. Even though NeoAgs are considered to be the ideal tumor-specific target antigens, their identification for personalized therapy presents significant challenges. These challenges include (i) the lack of evidence regarding the immunogenicity of the target peptide and/or TA presentation, (ii) a restricted comprehension of the exact binding characteristics of most human leukocyte antigen (HLA) class I and class II molecules with NeoAg peptides, and (iii) the prevalence of non-essential passenger or “branch” mutations in comparison to driver mutations, such as KRAS or TP53, which exhibit more “truncal” features that make them more attractive as NeoAg targets [88,89].

HLA class I peptide-binding prediction methods can generate a substantially higher number of potential neoantigen peptides than can be included in a multiepitope peptide vaccination protocol based on mutational profiling [90]. Neoantigens are a promising option for immunotherapy as they are not produced in healthy tissues and are not subject to thymic central tolerance mechanisms, which prevent the development of autoimmunity. These antigens have long been recognized as important targets for tumor therapy [91], and their significance has increased due to the availability and cost-effectiveness of next-generation sequencing [92,93,94]. Passenger mutations, which are usually not relevant for tumor growth, account for the vast majority of neoantigens. Nonetheless, they can also be highly immunogenic. Therefore, an individualized approach that selects target neoantigens based on the mutational profile of a patient’s tumor can utilize a broader range of neoantigens and tailor treatment to those with the highest potential for immunogenicity in that patient.

#### 5.1.1. Target Selection and Validation

In the clinical oncology field, identifying vaccine targets from small tumor samples is a critical task. With the advancement of next-generation sequencing technologies, it has become possible to target particular tumor mutations in cancer patients quickly and in cost-effective ways [95]. Although whole exome sequencing (WES) data from a cancer sample and sequencing data from identical normal cells can help identify targetable mutations in a patient, there are limitations. This is especially true when using tumor samples obtained from metastatic sites, chosen for therapeutic convenience. The presence of intra- and intertumoral heterogeneity is the primary reason for these limitations [96,97]. The presence of intra-tumor clonal heterogeneity and tumor growth are significant factors in the evolution of tumors. This evolution can result in subclones that are resistant to selection pressures, such as the immune response of the host [98].

Studies suggest that immunotherapy can modify the range of neoepitopes produced by cancer cells, leading to a decrease in the expression of highly expressed NeoAgs. Studies suggest that immunotherapy can modify the range of neoepitopes produced by cancer cells, leading to a decrease in the expression of highly expressed neoantigens. As a result, the immune system’s ability to identify and attack tumors is reduced, leading to immune evasion [99,100,101]. However, customized vaccination focusing on multiple neoepitopes rather than a single antigen can help to alleviate these issues. Due to the clonal evolution of tumors, there exists regional or geographic heterogeneity as well as clonal heterogeneity within tumors [102].

#### 5.1.2. Identifying Immunogenic Neoantigens

There were several broad similarities based on computational algorithm methods used for neoantigen identification and prioritization employed in investigations of immunotherapy [95,103,104]. To detect tumor-specific mutations, i.e., neoantigens, tumor biopsy samples and normal tissue samples were collected from the subject of interest. These samples were then subjected to whole exome sequencing to evaluate the DNA of both the tumor and the germline [95].

Depending on the type of tumor, a variety of tumor-specific mutations can be identified, but not all of these mutations produce neoepitopes that are recognizable by the immune system due to HLA restriction [105]. Computational approaches have been useful in identifying MHC molecules, such as MHC I-binding epitopes, that are more likely to elicit CD8+ T-cell responses [106]. Multiple algorithms have been developed to predict MHC I-presented epitopes, and mass spectrometry (MS) studies have been employed to improve prediction algorithms based on peptides eluted from MHC [107,108]. More research is needed to fully understand the mechanisms that govern neoantigen expression, presentation, and immunogenicity. The position of a mutant residue inside the peptide-binding groove, according to data, can aid in the development of neoantigens [109].

The effectiveness of therapeutic cancer vaccines is now being improved by enhancing tumor specific CD4^+^ T-cell responses. As previously mentioned, prediction algorithms are constantly improving, but other factors may impact the immunogenicity of the projected epitopes. Examples of these patterns include gene expression, RNA splicing, proteosomal processing, and most significantly, peptide loading and presentation by MHC [95]. Furthermore, due to T cells’ capacity to distinguish them from self-epitopes, NeoAg sequences that are identical to pathogen-derived epitopes may exhibit higher immunogenicity.

The incorporation of computational approaches in neoantigen-based therapies is expected to enhance their effectiveness, and the consideration of various parameters will result in more potent therapeutic T-cell vaccines [110]. There are different computational methods that can be used to induce neoantigen-specific immune responses. One of these methods involves taking autologous APCs, typically DCs, from a patient and exposing them to tumor lysates in vitro before re-injecting them into the patient. This approach does not require sequencing or computational analysis to identify patient-specific neoantigens [110]. However, tumor antigens are less likely to be immunogenic due to the presence of a larger quantity of nonimmunogenic self-antigens. This may limit the potential of relevant neoepitopes to elicit immunological responses. DC-based immune systems can be used to identify these neoepitopes (neoantigens), leading to increased immunization and activation of T cells.

## 6. DC-Based Immunotherapy in Lung Cancer

Lung cancer remains the most prevalent type of cancer-associated mortality worldwide. In an effort to explore potential immunotherapeutic strategies for various cancers, DCs have been studied due to their distinct ability to elicit and regulate T-cell responses. However, the course of treatment is determined by the tumor’s stage at the time of diagnosis and may include early-stage surgery, radiation, and chemotherapy, as well as palliative care for metastatic cancer. Despite improvements in cancer treatment, the outlook for patients remains poor, with a projected 5-year survival rate of only 18% [111]. Cancer vaccines present a specialized collection of antigenic targets to the immune system of the patient, resulting in a highly focused immune response against the tumor. The efficacy of such immunizations in activating a patient’s T cells is dependent on the level of local DC activation. However, advanced cancer patients often experience compromised DC activation, which can negatively impact the effectiveness of cancer vaccines [112,113,114,115,116].

**Figure 3:**
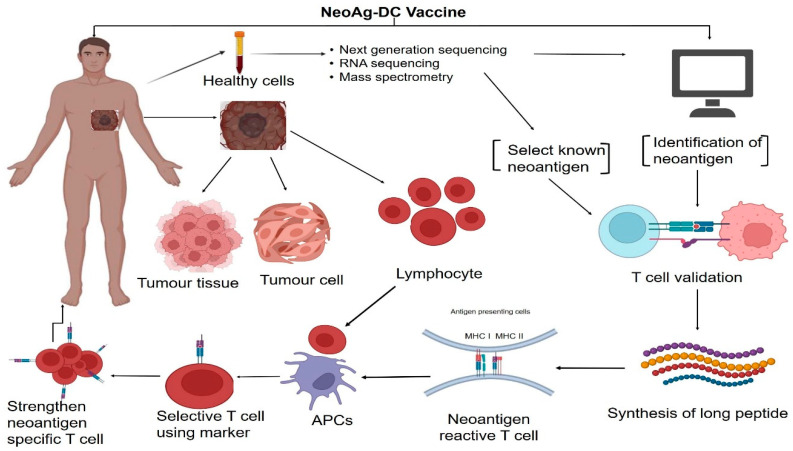
Schematic illustration of NeoAg lung cancer immunotherapy. The NeoAgs are isolated from lung cancer patient blood cells and tumor tissue. In silico methods identify major population NeoAgs. Immunotherapies like cancer vaccines use NeoAgs. Peptides, DNA, RNA, and DCs make up these cancer vaccines. In silico-generated NeoAgs are given to lung cancer patients, but personalized NeoAgs are given to the same patient. Cytokines, monoclonal antibodies against CD3 and CD28, and other reagents induce T-cell proliferation from a patient’s peripheral blood or tumor tissues. Co-culturing T cells with primed APCs and genetically engineering immune cells with TCRs or CARs produces NeoAgs-specific T lymphocytes. After T-cell expansion, lymphodepleted patients receive T-cell products to stimulate an immune response against tumors.

Ex vivo-produced APCs, such as DC-based vaccines, represent one of the most advanced forms of cancer immunotherapy that obviates the need for endogenous APCs from patients [117]. DCs were first identified by Ralph Steinman as highly effective APCs in 1973 [118]. DCs have a critical function in the activation, control, and development of immune responses that target tumors [16,118,119]. DCs can be found in all organs, and they continually scan their environment for danger signals and antigens, including those that are released during cancer formation. DCs are unique in their capacity to create new immune responses by breaking down acquired antigens into peptides and displaying them to inactivated T cells in lymphoid tissues through MHC molecules [119] (Figure 3).

The established role of DC-based immunotherapy in lung cancer treatment remains uncertain. For a DC vaccine approach, two key factors significantly affect its effectiveness: the careful choice of the appropriate DC subset and the selection of the right maturation state for the DCs. DC subsets, including conventional type I DCs (cDC1s), conventional type II DCs (cDC2s), plasmacytoid DCs (pDCs), and monocyte-derived dendritic cells (Mo-DCs) [120], have unique functions and impact immune responses differently. The decision on which DC subset to use depends on the specific objectives of the vaccine.

While in the field, cDC1s have been shown to be excellent at cross-presenting Ags to CD8+ T cells, recent publications also highlight that cDC2s could induce T-cell responses [121]. As such, other DC types have recently been assessed in clinical trials [122], though not yet in lung cancer. A series of non-randomized clinical studies utilizing DC immunotherapy have been conducted since the early 2000s. Despite the small sample sizes and divergent methodologies utilized in each study, the landscape of lung cancer treatment has undergone significant transformation during this period. Additionally, the maturation state of DCs significantly affects their ability to activate T cells and initiate immune responses [123]. DCs can be either immature or mature, and their maturation state influences how effectively they present antigens [123,124]. Mature DCs are skilled at presenting antigens to T cells and triggering a robust immune response [120]. However, the timing and degree of maturation are critical considerations. Premature or excessive maturation may lead to immune tolerance rather than activation [125]. Hence, careful thought about the timing and conditions that promote the appropriate maturation state is vital for maximizing the vaccine’s efficacy. Furthermore, optimizing the success of DC-based vaccines requires thoughtful consideration of both the specific DC subset chosen and the maturation state of the DCs. These factors ensure that the vaccine induces the desired immune response, establishing it as a powerful tool in therapeutic interventions and immunization strategies.

Presently, DC-based immunotherapy stands out as one of the most efficacious modalities available. Accordingly, this review aims to classify DC-based immunotherapy for lung cancer into four distinct subtypes [121,122,126].

### 6.1. Dendritic Cell-Based Therapy in NSCLC

The initial investigations in this area focused on individuals with metastatic or recurrent cancer who exhibited abnormal or elevated levels of carcinoembryonic antigen (CEA) in their serum [127]. CEA is a glycoprotein that plays a role in intercellular adhesion and is located in the membrane, with increased levels observed in various malignancies, such as NSCLC. 

Twelve patients diagnosed with either CRC or NSCLC were enrolled in a study where they received the Flt3 ligand, a hematopoietic growth factor known to increase DCs in vivo. Following this treatment, the patients underwent peripheral blood leukapheresis [128]. Subsequently, DCs were isolated and enriched with a supplementary protein referred to as KLH, which is not only capable of monitoring the immunological responses elicited by therapy but can also be utilized for the same purpose. Moreover, the DCs were also loaded with a nonapeptide synthesized from the CEA-specific HLA-A0201 peptide [128].

A total of 109 antigen-exposed DCs were intravenously (IV) administered to the patients, resulting in moderate diarrhea (5/12), self-limiting rigors, and fever (7/12) as the most common adverse effects. Of the twelve patients, seven exhibited a CEA-specific immune response following vaccination. One patient experienced a mixed response, while two demonstrated stable disease (SD), and two experienced significant tumor shrinkage. Furthermore, clinical responses were found to be associated with the expansion of CD8+ T cells. Itoh and Ueda et al. adopted a similar CEA-targeted DC immunization technique [128,129].

Another study conducted comprised 10 HLA-A24 patients with advanced lung or digestive tract cancer expressing CEA652. DCs were produced from peripheral blood mononuclear cells (PBMCs) by mobilizing plastic-adherent monocytes with granulocyte colony-stimulating factor (G-CSF) in the presence of granulocyte/macrophage colony-stimulating factor (GM-CSF) and interleukin 4 (IL-4). The immature DCs contained the CEA652 nonapeptide. During the immunization period, patients received numerous injections administered via the intradermal and subcutaneous routes. In addition, seven patients received adjuvant interferon alpha (IFN-α) and TNF-α twice weekly. The vaccine was well tolerated, and no severe side effects were reported. The delayed-type hypersensitivity skin test showed that two patients exhibited a positive response to peptide-pulsed DCs following vaccination, while none of the patients displayed such a response before vaccination [128]. Most of patients who experienced clinically beneficial treatment showed cutaneous responses to CEA652-pulsed DCs and in vitro CTL responses to CEA652 peptide. The study emphasizes how important it is for T cells, specifically those targeting the CEA652 peptide, to stick around for a while to keep fighting off tumors effectively. But, to really understand how long they hang in there and how that affects patients in the long run, more research is necessary. As for overall survival (OS) and progression-free survival (PFS), while the study did not dive into those specifics, it is promising to see that patients with a positive immune response showed stable disease. This suggests that revving up the immune system could potentially improve clinical outcomes, but we will need bigger studies with longer follow-ups to confirm [128].

In several studies, CEA has been identified as the antigen of choice for DC-based active immunotherapy. The administration of CEA resulted in the elicitation of both CEA- and tumor-specific cytotoxic T-lymphocyte responses, with the latter exhibiting superior potency. Further investigations into MUC1-targeted immunization strategies in NSCLC demonstrated similar clinical effectiveness [113,130].

A study involved 16 patients with stages IA-IIIB NSCLC who received autologous DC vaccinations that were matured using DC/T cell-derived maturation factor (DCTCMF) [131]. Seven patients had undergone surgical resection (stage I/II) either with or without adjuvant therapy, and the remaining seven patients with unresectable stage III disease were managed solely with chemoradiation. The researchers used pulsed autologous DCs combined with apoptotic bodies obtained from an allogeneic NSCLC cell line. This allogeneic NSCLC cell line upregulated several proteins such as Her2/neu, CEA, WT1, Mage2, and survivin, and did not involve any maturational stimulation. The resultant immunological responses were noted in four out of seven individuals with unresectable stage III disease, six out of seven patients who had undergone surgical resection for stage I/II cancer, and all three surgically resected patients who had undergone adjuvant chemotherapy and radiotherapy. Notably, no adverse events were recorded.

Among the surgically resected patients, only one out of seven experienced a relapse, whereas four out of seven individuals with stage III cancer experienced disease progression. Interestingly, three out of five patients with advancing illness displayed no discernible immune response. These findings imply that DCs in an immature state that were pulsed with apoptotic tumor cells have a biological activity that is comparable to that of DCs matured with DCTCMF and administered using a similar clinical regimen [131]. The study did not explicitly report the duration of T-cell persistence post-vaccination. Vaccine-induced immune responses, including T-cell responses, commonly wane over time without continuous stimulation. While the study emphasized immunological responses, it lacked detailed data on OS and PFS for both surgically resected (stage I/II) and unresectable stage III patients, making it difficult to assess the vaccine’s impact on these clinical outcomes.

The study (NCT02956551) involving 12 patients who received personalized neoantigen-pulsed DC vaccines for advanced lung cancer showed promising results. The treatment included 85 vaccine doses, averaging five doses per person, with each patient receiving between 12 and 30 neoantigens. Adverse events were mostly mild to moderate (grade 1–2), and there were no treatment delays due to toxicity. The treatment achieved an objective effectiveness rate of 25% and a disease control rate of 75%. The median PFS was 5.5 months, while the median OS was 7.9 months, indicating positive outcomes for lung cancer patients [63]. Nonetheless, these clinical studies have demonstrated limited efficacy and anecdotal outcomes. As a result, alternative strategies have been devised to overcome these substantial obstacles.

### 6.2. DC/Cytokine-Induced Killer (CIK) Cells Therapy in NSCLC

Numerous clinical trials have been conducted in recent years to investigate the efficacy of autologous DCs and CIK cells in the treatment of NSCLC [132]. In the study, DC–CIK immunotherapy involves the activation and expansion of CIK cells by DCs, which are capable of recognizing and killing tumor cells. The study found that DC–CIK immunotherapy significantly improved the PFS and OS in NSCLC patients compared to control therapies. The hazard ratio (HR) for PFS was 0.528 (95% confidence interval: 0.390–0.715), and for OS, it was 0.619 (95% CI: 0.487–0.786). Additionally, the disease control rate (DCR) was significantly improved with DC–CIK immunotherapy, with a relative risk (RR) of 1.250 (95% CI: 1.058–1.477). However, the objective response rate (ORR) did not show a significant improvement with DC–CIK immunotherapy (RR: 1.190, 95% CI: 0.561–2.526) [132].

CIK cells, a non-MHC-restricted subset of natural killer T lymphocytes, exhibit potent cytolytic activity against cancerous cells and can be rapidly expanded in vitro [133]. In DC/CIK therapy, mononuclear cells obtained through leukapheresis are first converted into DCs, which are subsequently loaded with antigens in a conventional GM-CSF/IL-4-supplemented medium. Meanwhile, CIK cells are generated by culturing PBMCs in a medium supplemented with anti-CD3 antibody, recombinant human IL-1α, IFN-γ, and IL-2 [134]. A range of disease scenarios have been investigated using DC/CIK cell therapy, including adjuvant therapy for resectable cancer, first-line therapy for patients with stages IIIB and IV, and maintenance therapy after first-line chemotherapy [135,136,137].

In a particular study, DC–CIK therapy was administered to 135 patients with advanced NSCLC, either in combination with chemotherapy or as a monotherapy [138]. The median progression-free survival (PFS) was 5.7 months, and the median overall survival (OS) was 17.5 months. The 1-year PFS and OS rates were 29.4 and 58.8 percent, respectively, with a significant improvement observed in the combination group compared to the monotherapy groups. The quantity of infused DC–CIK cells was positively correlated with clinical efficacy, and both DC–CIK plus chemotherapy and the quantity of infused CIKs were independent predictors of PFS and OS. A significant alteration in T-cell subsets was observed across all groups, with CD8+CD28+ and CD8+CD28- T cells showing significant changes. CD3+ T cells increased, while CD3-CD16+CD56 T cells decreased in the chemotherapy plus immunotherapy and immunotherapy-alone groups. The clinical activity observed in this trial is encouraging [138]. While the study did not directly address the duration of T-cell persistence after vaccination, the combination of DC–CIK with CT showed promising results in terms of PFS and OS in advanced NSCLC patients. Further research is needed to explore the optimal vaccine strategies and their impact on T-cell persistence and long-term treatment outcomes in cancer immunotherapy [138].

In recent years, the unique concept of combining thoracic radiation (TRT) or chemoradiotherapy (CRT) with DC/CIK cell therapy has emerged. The underlying principle is that radiation-killed tumor cells release TAAs/TSAs and “danger-associated molecular patterns,” which may attract DCs to initiate TAA/TSA-specific CD8+ T-cell responses, thus enhancing objective responses and improving survival outcomes [136,139]. However, despite their potential benefits, these methods have certain limitations.

### 6.3. Activated Killer T Cells (AKT)—DC Therapy in NSCLC

Unlike the therapies mentioned earlier, a unique form of adoptive immunotherapy employs autologous activated killer T cells and DCs (AKT–DC) obtained from the tissue cultures of tumor-draining lymph nodes in primary lung tumors. According to Kimura and his team, these lymph nodes can be a reliable source of mature DCs and killer T cells that are specific to the patient’s tumor cells when incubated with low doses of IL-2. For the study, 31 patients were eligible, but three were excluded due to patient refusal after receiving 1–2 rounds of immunotherapy. Out of the 28 treated cases, a total of 313 immunotherapy courses were administered. Common toxicities included fever (78.0%), chills (83.4%), fatigue (23.0%), and nausea (17.0%) on the day of cell transfer. The survival rates at 2 and 5 years were 88.9% (95.9–81.9; 95% confidence interval) and 52.9% (76.4–29.4; confidence interval), respectively. They also demonstrated that the T-cell proliferation in vitro can persist for a duration of up to two months [140]. An investigation of the effectiveness and safety of chemo-immunotherapy employing these AKT–DCs in postoperative N2 NSCLC patients was performed in a phase II trial based on this mechanism [140].

Twenty-eight participants received four cycles of chemotherapy every two months for two years along with AKT–DC immunotherapy. The most frequently observed adverse events were fever and chills. A phase III randomized controlled trial was conducted by the same team to evaluate the effectiveness of adjuvant chemo-immunotherapy with AKT–DC targeting residual micro metastases in 103 patients with resected NSCLC. The two-year and five-year OS rates were found to be 88.9% and 52.9%, respectively [141]. In another study, 103 patients with post-surgical lung cancer were randomly assigned to receive AKT and DCs administered with CT or monotherapy. Patients in the immunotherapy arm (group A) received chemo-immunotherapy, while those in the control arm (group B) received chemotherapy. The study involved administering activated AKT–DCs as a treatment. Group A, which received chemo-immunotherapy, had 2 and 5 year OS rates of 96.0% and 69.4%, respectively, while group B, which received chemotherapy, had rates of 64.7% and 45.9%, respectively. A multivariable analysis showed a risk ratio of 0.439%. Group A had 2 and 5 year recurrence-free survival rates of 70.0% and 57.9%, respectively, compared to 43.1% and 31.4% for group B. An immunological examination of cell surface markers in regional lymph nodes of immunotherapy recipients revealed an increased ratio of CD8^+^/CD4^+^ T cells in survivors. The study suggests that adoptive cellular immunotherapy may be a beneficial adjunct to surgery for NSCLC patients. However, the study has limitations, such as the absence of a heterogeneous population study, even though the data demonstrate clinical significance for patients with lung cancer [141].

### 6.4. DC-Based Therapy in SCLC

Studies in the treatment of SCLC have shown limited responses to immune checkpoint suppression compared to NSCLC, likely due to the immunological and pathological differences between the two types of cancer [142,143]. Additionally, there are not many SCLC immunotherapy trials using DCs. One approach that has been investigated involves using DCs transduced with an adenovirus expressing p53 (Ad. p53) in patients with advanced SCLC [144,145]. However, about 90% of SCLC patients have p53 tumor suppressor gene mutations that regulate cell division and proliferation [145].

In a phase II study, patients with extensive-stage SCLC who had undergone chemotherapy were given DCs transfected with wild-type TP53 (vaccine) [144,145]. The vaccinations were administered with a 2-week interval for the first three doses and a 4-week interval for the subsequent doses. Each vaccination had a maximum dose of 5 × 10^6^ cells [144]. Notably, it indicates that T-cell proliferation in vitro was observed to persist for up to 2–4 weeks following vaccination.

Participants were randomly assigned to one of three arms: arm A (observation), arm B (vaccine alone), or arm C (1:1:1) (vaccine plus all-trans-retinoic acid). The study used a 2-stage Simon minimax design, with 55 patients treated in stage 1 (18 in arm A, 20 in arm B, and 17 in arm C) and 14 in stage 2 (only in arm C), out of the 69 patients enrolled (median age 62 years, performance status 0/1). Although the vaccine was safe, with mostly grade 1/2 toxicities, one patient in arm B reported grade 3 fatigue and eight patients in arm C experienced grade 3 toxicities. Despite the vaccine not improving the OS rate to second-line chemotherapy, it displayed a safe profile and therapeutic immunological potential, suggesting that it could be combined with other immunotherapeutic drugs. In a subsequent randomized phase II trial, the same team enrolled 69 patients with severe SCLC who were responsive to treatment or had non-progressive disease after first-line conventional chemotherapy [146,147].

In a previous trial, the Ad. p53-DC vaccine was found to be safe and able to induce a cytotoxic T cell response in 20–40% of patients with advanced SCLC, but this did not translate into significant clinical benefit. However, a subsequent trial showed a higher-than-expected response rate to second-line paclitaxel, providing promise for combination treatments that combine chemotherapy with immunotherapy to improve efficacy [148]. Another study involved repeated vaccination at 2-week intervals in 29 SCLC patients. The evaluation of clinical response was conducted to assess the effects of vaccination and subsequent chemotherapy, where 57.1% of patients displayed p53-specific T-cell responses to immunization. Notably, a significant proportion of patients (61.9%) exhibited objective clinical responses immediately following vaccination, and this response was highly correlated with the immunological response induced by vaccination. These findings provide clinical evidence supporting a new paradigm in cancer immunotherapy, wherein vaccination may prove more effective in combination with chemotherapy rather than as a standalone modality [145].

## 7. Limitations of Vaccines Based on Neoantigens

Immunotherapeutic DC vaccines for lung cancer have several limitations. One of the limitations of the vaccination protocol was the utilization of immature DCs. Despite their role as potent inducers of immunological tolerance, immature DCs demonstrated low efficacy in generating immune responses, which is not desirable in the context of cancer immunotherapy. To address this issue, it is possible to expose DCs to a diverse array of chemical combinations to promote maturation. Nevertheless, due to concerns regarding stability, not all of these combinations can be readily incorporated into a clinical-grade production process [148]. An additional limitation, which has received insufficient attention, pertains to the injection method utilized for DCs. Intradermal or subcutaneous (s.c.) injections have been employed in several clinical trials due to their safety and ease of use. However, the bulk of DCs administered via these methods tend to remain at the injection site, and do not effectively migrate to lymph nodes that are abundant in T cells [149].

The optimal dosage and injection schedule for DC therapy remain unknown. As the mechanism of action of DC therapy is indirect and immunological responses are insufficient surrogates for clinical outcomes, appropriate dose–effect models are yet to be established [148]. Furthermore, there are challenges in scaling up autologous cell treatments like DC-based immunotherapies to cater to a larger number of patients, requiring manufacturers and healthcare professionals to address affordability concerns [21]. In cellular therapy, generating an immune response against tumors is challenging due to toxicity, the lack of TSAs, and the hostile TME. By disrupting the immune suppressive mechanisms orchestrated by these cells, the TME can be reprogrammed to support anti-tumor immune responses and enhance the effectiveness of cancer vaccines.

NeoAgs have garnered attention as promising TSAs, but accurate prediction is limited due to genetic heterogeneity, including variations in somatic mutations among cancer types, individuals, and tumor subclones. Tumors employ mechanisms, such as NeoAg loss, altered antigen presentation, and an immunosuppressive TME, to evade NeoAg-based immunotherapies [148].

## 8. Conclusions

In recent years, significant improvements have been made in our understanding of the molecular and cellular pathways involved in the spontaneous recognition and elimination of both premalignant and malignant cells by the immune system. The advent of immunotherapy has brought about a paradigm shift in lung cancer treatment, and this field is continually evolving. This knowledge has been crucial in the development of various therapeutic approaches that target the restoration of anti-cancer immune surveillance, such as DC-based immunization. Immunotherapy using DCs has been shown to be a safe and well-tolerated treatment that can induce anti-tumor immune responses in patients with lung cancer. Combining DC-based immunotherapy with other cancer treatments, such as chemotherapy, radiation, and/or checkpoint blockade, has the potential to increase their effectiveness. Choosing antigens based on neoepitopes expressed by tumor cells may stimulate immune responses and lead to clinical responses. These concepts are currently being tested in clinical trials, and their results are eagerly awaited. Overcoming challenges such as establishing the appropriate dose, frequency, and duration of treatment, enhancing target antigen selection, and identifying biomarkers for early recognition of potential responders are imperative for the future advancement of DC therapy. Ultimately, the identification of the most synergistic combination regimen with tumor antigens (NeoAgs) is crucial for achieving long-term disease control and improving the survival of patients with this life-threatening ailment.

## 9. Future Perspectives

Future investigations could focus on studying how antigen characterization and DC maturation impact immunological responses. Another important aspect to consider is the immunosuppressive cytokine release and tumor escape from immune surveillance via antigen and MHC downregulation. Although cellular treatment is promising, it is challenging due to the lack of TSAs, toxicity, and a hostile TME. To reduce the hostility of the TME and enhance the efficacy of cancer vaccines using neoantigens as DC vaccines, a multifaceted approach is essential. First, optimizing the selection and presentation of neoantigens is crucial. Advanced genomic and proteomic techniques can identify tumor-specific neoantigens that are more likely to elicit a strong immune response. Coupling these antigens with potent adjuvants can further enhance the immunogenicity of DC vaccines, making them more effective at initiating and sustaining anti-tumor immune responses. And, to modulate the hostility of the TME and enhance the efficacy of cancer vaccines, future strategies can focus on targeting immune-suppressive cells (i.e., Tregs and MDSCs) within the TME. Clinical trials, such as NCT03927105, NCT01596751, NCT03153410, and others, are investigating drugs specifically designed to counteract these cells to improve treatment outcomes. Modulating the TME to overcome its immunosuppressive nature is another critical strategy. The TME often harbors cells and factors that suppress immune responses, such as regulatory T cells (Tregs) and myeloid-derived suppressor cells (MDSCs), along with immunosuppressive cytokines. Targeting these components through the use of checkpoint inhibitors, cytokine blockers, or cell depletion strategies can help in creating a more favorable environment for the DC vaccine-induced immune response to act against the tumor cells.

Additionally, dosage, immunization regimens, and modes of delivery are also difficult to optimize in this field. Although convenient and safe intradermal or subcutaneous injections are frequently utilized in clinical trials, a large fraction of given DCs remain localized at the injection site instead of moving to T cell-rich lymph nodes. Radiation therapy, which stimulates DC migration to lymph nodes, can be used in concert with other therapies to overcome this obstacle. DCs can then interact with T cells to start adaptive immunological responses.

Another approach is to overcome these limitations, and extracellular vesicles (EVs) have emerged as a promising candidate. Research has demonstrated that EVs derived from tumor cells can serve as a promising vaccination strategy that boosts DC maturation and the presentation of NeoAgs. This allows for the cross-presentation of these NeoAgs to immune cells. Additionally, EVs derived from DCs themselves can act as presenting units for neoantigens, offering an alternative framework for neoantigen-focused cancer vaccines. Recent studies have demonstrated that EV-based vaccination can modulate the TME and systemic immune responses, thus transforming a “cold” tumor into a “hot” tumor. This approach may enable the administration of neoantigen-based cancer vaccines orally. The field of anti-cancer vaccines is poised for evolution, with a focus on personalization through NeoAg targeting. While DC-based immunotherapy remains significant, newer modalities such as mRNA/NeoAg-loaded LNPs have gained attention. LNPs, tailored to individual tumor profiles, hold promise for more effective cancer immunotherapy. In summary, anti-cancer vaccine development is entering an exciting phase of diversification and innovation, offering great potential for enhanced personalized cancer treatment.

## Figures and Tables

**Table 1:** Tumor-specific antigens (TSAs) and tumor-associated antigens (TAAs) are the two classifications of tumor antigens outlined in this table. There are several distinctions between TSAs and TAAs. TSAs are exclusively found in tumor cells and do not exhibit immune evasion. They also do not elicit autoimmune responses and pose minimal risk for vaccine development. Conversely, TAAs differ from TSAs in that they may show immune escape mechanisms and have a higher likelihood of triggering autoimmunity.

Properties	Tumor-Associated Antigens (TAAs)	Tumor-Specific Antigens (TSAs)
Normal cell	Yes	No
Tumor cell	Yes	Yes
Immune escape	Yes	No
Autoimmunity	Yes	No
Risk of vaccines (previous experimental studies)	Yes (High)	No (Minimum)
Examples	p53, Ras, Bcr-Abl (case-specific mutated neoantigen)	HPV E6, E7, Her2/neu, telomerase, survivin, Gp100, tyrosinase

**Table 2:** The table shows the most recent types of immune blockades used in cancer immunotherapy, with a focus on how their ligands work and how they can be used to treat lung cancer. It gives an outline of the different kinds of immune checkpoint inhibitors that have been made to boost the body’s natural immune response against cancer cells.

Immune Blockade	Ligand	Mechanism of Action	Lung Cancer Indication	Research Paper Citation
PD-1	PD-L1	PD-1 inhibition blocks the interaction between PD-1 on T cells and PD-L1 on cancer cells, thereby restoring T-cell function and promoting anti-tumor immune responses.	Non-small cell lung cancer	Reck et al., 2016 [57]
CTLA-4	B7	CTLA-4 inhibition enhances T-cell activation and proliferation by blocking the interaction between CTLA-4 on T cells and B7 on antigen-presenting cells, leading to increased anti-tumor immune responses.	Non-small cell lung cancer	Hellmann et al., 2018 [58]
TIM-3	Galectin-9, CEACAM1	TIM-3 inhibition prevents the interaction between TIM-3 on T cells and its ligands on cancer cells, leading to improved T-cell function and enhanced anti-tumor immune responses.	Non-small cell lung cancer	Harding et al., 2021 [54]
LAG-3	MHC class II	LAG-3 inhibition blocks the interaction between LAG-3 on T cells and MHC class II on antigen-presenting cells, leading to enhanced T-cell activation and anti-tumor immune responses.	Non-small cell lung cancer	Eng et al., 2019 [59]
TIGIT	CD155, CD112	TIGIT inhibition prevents the interaction between TIGIT on T cells and its ligands on cancer cells, leading to improved T-cell function and enhanced anti-tumor immune responses.	Non-small cell lung cancer	Hung et al., 2018 [60]
VISTA	PSGL-1, C-type lectin domain family 1 member B	VISTA inhibition blocks the interaction between VISTA on cancer cells and its ligands on T cells, leading to enhanced T-cell activation and anti-tumor immune responses.	Non-small cell lung cancer	Qin et al., 2019 [61]

**Table 3:** Ongoing clinical trials testing DC immunotherapy in patients affected by lung cancer (NSCLC and SCLC) (https://clinicaltrials.gov/).

	NCT Number	Study Title	Conditions	Interventions	Study Phases	Start Date	Completion Date	OS and PFS (Months)	References
**DC-based immunotherapy in NSCLC**	NCT02956551	Personalized DC Vaccine for Lung Cancer	Carcinoma, NSCLC	BIOLOGICAL: DC vaccine	PHASE1	November 2016	January 2020	OS = 7.9; PFS = 5.5	[63]
	NCT00322452	First Line IRESSA^TM^ Versus Carboplatin/Paclitaxel in Asia	NSCLC	DRUG: Gefitinib| DRUG: Carboplatin| DRUG: Paclitaxel	PHASE3	March 2006	June 2010	NA	[64]
	NCT03546361	CCL21-Gene Modified DC Vaccine and Pembrolizumab in Treating Patients With Stage IV NSCLC	NSCLC |Stage IV Lung Cancer AJCC v8|Stage IVA Lung Cancer AJCC v8|Stage IVB Lung Cancer AJCC v8	BIOLOGICAL: Autologous DC-Adenovirus CCL21 vaccine |BIOLOGICAL: Pembrolizumab	PHASE1	July 2019	June 2025	NA	[65]
	NCT05195619	Personalized DC Vaccines in NSCLC	NSCLC	BIOLOGICAL: Autologous DC vaccine loaded with personalized peptides |DRUG: Low dose cyclophosphamide	PHASE1	December 2021	September 2024	OS = 2; PFS = 2	[66]
	NCT00322452	First Line IRESSA^TM^ Versus Carboplatin/Paclitaxel in Asia	NSCLC	DRUG: Gefitinib, Carboplatin, Paclitaxel	PHASE3	March 2006	June 2010	OS = 18.1 (gefitinib) vs. 18.3 months (carboplatin/paclitaxel); PFS = 6.8	[67]
	NCT04147078	Personalized DC Vaccine for Postoperative Cancer	Gastric Cancer| Hepatocellular Carcinoma |NSCLC| Colon Rectal Cancer	BIOLOGICAL: DC vaccine subcutaneous administration	PHASE1	June 2019	June 2026	NA	[68]
**DC/CIK Cell Therapy**	NCT03987867	Study of Autologous CIK Cell Immunotherapy Combination With PD-1 Inhibitor and Chemotherapy in the Advanced NSCLC	NSCLC Cancer| First-line Treatment	BIOLOGICAL: CIK cell| BIOLOGICAL: Sintilimab injection| DRUG: Pemetrexed, Liposome Paclitaxel, Carboplatin	PHASE1	June 2019	June 2021	PFS = 19.3	[69]
	NCT03360630	Anti-PD-1 Alone or Combined With Autologous Cell Therapy in Advanced NSCLC	Lung Cancer| Neoplasms NSCLC	BIOLOGICAL: Anti-PD-1 plus DC–CIK|BIOLOGICAL: Anti-PD-1 alone	PHASE1|PHASE2	November 2016	June 2023	OS = 24	[70]
	NCT01871480	CIK Cell Transfusion Plus Gefitinib As Second Or Third-Line Treatment for Advanced Adenocarcinoma NSCLC	NSCLC	DRUG: Group A: Cytokine-induced killer cell + Gefitinib |DRUG: Group B: Gefitinib	PHASE2	May 2013	May 2016	OS = 3; PFS = 2	[71]
**AKT Therapy in NSCLC**	NCT01294306	MK2206 and Erlotinib Hydrochloride in Treating Patients With Advanced NSCLC Who Have Progressed After Previous Response to Erlotinib Hydrochloride Therapy	Adenosquamous Lung Carcinoma |Bronchioloalveolar Carcinoma| Large Cell Lung Carcinoma| Lung Adenocarcinoma| Recurrent NSCLC| SCLC	DRUG: Akt inhibitor MK2206|DRUG: Erlotinib hydrochloride |OTHER: Laboratory biomarker analysis |OTHER: Pharmacological study	PHASE2	February 2011	August 2015	PFS = 4.4; 4.6	[72]
**DC-based therapy in SCLC**	NCT02688686	Safety and Efficacy of DC–CIK in Patients With Advanced NSCLC Cancer With Bone Metastases	NSCLC With Bone Metastases	BIOLOGICAL: Genetically modified DCs + CIK BIOLOGICAL: Neoantigen-loaded DC vaccine	PHASE1|PHASE2	February 2016	NA	NA	[73]
	NCT03871205	Neoantigen-primed DC Vaccines Therapy for Refractory Lung Cancer	NSCLC| SCLC	BIOLOGICAL: Neoantigen-loaded DC vaccine	PHASE1	April 2019	December 2020	NA	[74]
	NCT03406715	Combination Immunotherapy-Ipilimumab-Nivolumab-DC p53 Vac—Patients With SCLC	SCLC| Lung Cancer| Relapsed SCLC	DRUG: Nivolumab| DRUG: Ipilimumab| BIOLOGICAL: DC-based p53 vaccine	PHASE2	March 2018	December 2023	OS = 3; PFS = 3	[75]
	NCT02956551	Personalized DC Vaccine for Lung Cancer	Carcinoma, NSCLC	BIOLOGICAL: DC vaccine	PHASE1	November 2016	June 2020	OS = 7.9; PFS = 5.5	[76]
	NCT03546361	CCL21-Gene Modified DC Vaccine and Pembrolizumab in Treating Patients With Stage IV NSCLC	NSCLC Carcinoma| Stage IV Lung Cancer AJCC v8|Stage IVA Lung Cancer AJCC v8|Stage IVB Lung Cancer AJCC v8	BIOLOGICAL: Autologous DC–Adenovirus CCL21 Vaccine| BIOLOGICAL: Pembrolizumab	PHASE1	July 2019	June 2025	NA	[65]
	NCT05195619	Personalized DC Vaccines in NSCLC	NSCLC	BIOLOGICAL: Autologous DC vaccine loaded with personalized peptides (PEP-DC vaccine)|DRUG: Low dose cyclophosphamide	PHASE1	December 2021	September 2024	OS = 2; PFS = 2	[66]

**Abbreviations:** DC, dendritic cell; NA, not available; NSCLC, non-small cell lung cancer; SCLC, small cell lung cancer; CIK, Cytokine-induced killer cells; CTL, Cytotoxic T lymphocytes; PEP, personalized peptides; AJCC, American Joint Committee on Cancer; v8, Version 8; CCL21, C-C Motif Chemokine Ligand 21; NCT, National Clinical Trial.

## Data Availability

Not applicable.

## References

[1] Sung H., Ferlay J., Siegel R.L., Laversanne M., Soerjomataram I., Jemal A., Bray F. (2021). Global Cancer Statistics 2020: GLOBOCAN Estimates of Incidence and Mortality Worldwide for 36 Cancers in 185 Countries. CA Cancer J. Clin..

[2] Rudin C.M., Brambilla E., Faivre-Finn C., Sage J. (2021). Small-cell lung cancer. Nat. Rev. Dis. Primers.

[3] Kaumaya P.T. (2020). B-cell epitope peptide cancer vaccines: A new paradigm for combination immunotherapies with novel checkpoint peptide vaccine. Future Oncol..

[4] Antonarelli G., Corti C., Tarantino P., Ascione L., Cortes J., Romero P., Mittendorf E., Disis M., Curigliano G. (2021). Therapeutic cancer vaccines revamping: Technology advancements and pitfalls. Ann. Oncol..

[5] Zhang Y., Zhang Z. (2020). The history and advances in cancer immunotherapy: Understanding the characteristics of tumor-infiltrating immune cells and their therapeutic implications. Cell. Mol. Immunol..

[6] Yang J., Zhang Q., Li K., Yin H., Zheng J.N. (2015). Composite peptide-based vaccines for cancer immunotherapy. Int. J. Mol. Med..

[7] Amara S., Tiriveedhi V. (2017). The five immune forces impacting DNA-based cancer immunotherapeutic strategy. Int. J. Mol. Sci..

[8] Coulie P.G., Van den Eynde B.J., Van Der Bruggen P., Boon T. (2014). Tumour antigens recognized by T lymphocytes: At the core of cancer immunotherapy. Nat. Rev. Cancer.

[9] Li L., Goedegebuure S., Gillanders W.E. (2017). Preclinical and clinical development of neoantigen vaccines. Ann. Oncol..

[10] Gatti-Mays M.E., Redman J.M., Collins J.M., Bilusic M. (2017). Cancer vaccines: Enhanced immunogenic modulation through therapeutic combinations. Hum. Vaccines Immunother..

[11] Tiptiri-Kourpeti A., Spyridopoulou K., Pappa A., Chlichlia K. (2016). DNA vaccines to attack cancer: Strategies for improving immunogenicity and efficacy. Pharmacol. Ther..

[12] Gasser M., Waaga-Gasser A.M. (2016). Therapeutic antibodies in cancer therapy. Adv. Exp. Med. Biol..

[13] Robson N.C., Hoves S., Maraskovsky E., Schnurr M. (2010). Presentation of tumour antigens by dendritic cells and challenges faced. Curr. Opin. Immunol..

[14] Takahashi Y., Harashima N., Kajigaya S., Yokoyama H., Cherkasova E., McCoy J.P., Hanada K.-i., Mena O., Kurlander R., Abdul T. (2008). Regression of human kidney cancer following allogeneic stem cell transplantation is associated with recognition of an HERV-E antigen by T cells. J. Clin. Investig..

[15] Savina A., Amigorena S. (2007). Phagocytosis and antigen presentation in dendritic cells. Immunol. Rev..

[16] Melief C.J. (2008). Cancer immunotherapy by dendritic cells. Immunity.

[17] Lin M.J., Svensson-Arvelund J., Lubitz G.S., Marabelle A., Melero I., Brown B.D., Brody J.D. (2022). Cancer vaccines: The next immunotherapy frontier. Nat. Cancer.

[18] Barbier A.J., Jiang A.Y., Zhang P., Wooster R., Anderson D.G. (2022). The clinical progress of mRNA vaccines and immunotherapies. Nat. Biotechnol..

[19] Bravo Montenegro G., Farid S., Liu S.V. (2021). Immunotherapy in lung cancer. J. Surg. Oncol..

[20] Saxena M., van der Burg S.H., Melief C.J.M., Bhardwaj N. (2021). Therapeutic cancer vaccines. Nat. Rev. Cancer.

[21] Lopes A., Vandermeulen G., Préat V. (2019). Cancer DNA vaccines: Current preclinical and clinical developments and future perspectives. J. Exp. Clin. Cancer Res..

[22] Occhipinti S., Sponton L., Rolla S., Caorsi C., Novarino A., Donadio M., Bustreo S., Satolli M.A., Pecchioni C., Marchini C. (2014). Chimeric Rat/Human HER2 Efficiently Circumvents HER2 Tolerance in Cancer PatientsChimeric HER2 Vaccines Overcome Cancer Patient’s T-cell Dysfunction. Clin. Cancer Res..

[23] Dong Y., Dai T., Wei Y., Zhang L., Zheng M., Zhou F. (2020). A systematic review of SARS-CoV-2 vaccine candidates. Signal Transduct. Target. Ther..

[24] Paston S.J., Brentville V.A., Symonds P., Durrant L.G. (2021). Cancer vaccines, adjuvants, and delivery systems. Front. Immunol..

[25] Nguyen-Hoai T., Pezzutto A., Westermann J. (2022). Gene gun Her2/neu DNA vaccination: Evaluation of vaccine efficacy in a syngeneic Her2/neu mouse tumor model. Methods Mol. Biol..

[26] Trimble C., Lin C.-T., Hung C.-F., Pai S., Juang J., He L., Gillison M., Pardoll D., Wu L., Wu T.-C. (2003). Comparison of the CD8+ T cell responses and antitumor effects generated by DNA vaccine administered through gene gun, biojector, and syringe. Vaccine.

[27] Shetty K., Ott P.A. (2021). Personal neoantigen vaccines for the treatment of cancer. Annu. Rev. Cancer Biol..

[28] Kranz L.M., Diken M., Haas H., Kreiter S., Loquai C., Reuter K.C., Meng M., Fritz D., Vascotto F., Hefesha H. (2016). Systemic RNA delivery to dendritic cells exploits antiviral defence for cancer immunotherapy. Nature.

[29] Lu D., Benjamin R., Kim M., Conry R., Curiel D. (1994). Optimization of methods to achieve mRNA-mediated transfection of tumor cells in vitro and in vivo employing cationic liposome vectors. Cancer Gene Ther..

[30] Sahin U., Derhovanessian E., Miller M., Kloke B.-P., Simon P., Löwer M., Bukur V., Tadmor A.D., Luxemburger U., Schrörs B. (2017). Personalized RNA mutanome vaccines mobilize poly-specific therapeutic immunity against cancer. Nature.

[31] Matsushita H., Vesely M.D., Koboldt D.C., Rickert C.G., Uppaluri R., Magrini V.J., Arthur C.D., White J.M., Chen Y.-S., Shea L.K. (2012). Cancer exome analysis reveals a T-cell-dependent mechanism of cancer immunoediting. Nature.

[32] Larocca C., Schlom J. (2011). Viral vector-based therapeutic cancer vaccines. Cancer J..

[33] Hollingsworth R.E., Jansen K. (2019). Turning the corner on therapeutic cancer vaccines. Npj Vaccines.

[34] Wilson N.S., Villadangos J.A. (2005). Regulation of antigen presentation and cross-presentation in the dendritic cell network: Facts, hypothesis, and immunological implications. Adv. Immunol..

[35] Terbuch A., Lopez J. (2018). Next generation cancer vaccines—Make it personal!. Vaccines.

[36] Liu Z., Roche P.A. (2015). Macropinocytosis in phagocytes: Regulation of MHC class-II-restricted antigen presentation in dendritic cells. Front. Physiol..

[37] Berzofsky J.A., Terabe M., Oh S., Belyakov I.M., Ahlers J.D., Janik J.E., Morris J.C. (2004). Progress on new vaccine strategies for the immunotherapy and prevention of cancer. J. Clin. Investig..

[38] Steinman R.M., Dhodapkar M. (2001). Active immunization against cancer with dendritic cells: The near future. Int. J. Cancer.

[39] Nestle F.O. (2000). Dendritic cell vaccination for cancer therapy. Oncogene.

[40] Hsu F.J., Benike C., Fagnoni F., Liles T.M., Czerwinski D., Taidi B., Engleman E.G., Levy R. (1996). Vaccination of patients with B-cell lymphoma using autologous antigen-pulsed dendritic cells. Nat. Med..

[41] Donninger H., Li C., Eaton J.W., Yaddanapudi K. (2021). Cancer vaccines: Promising therapeutics or an unattainable dream. Vaccines.

[42] Hailemichael Y., Dai Z., Jaffarzad N., Ye Y., Medina M.A., Huang X.-F., Dorta-Estremera S.M., Greeley N.R., Nitti G., Peng W. (2013). Persistent antigen at vaccination sites induces tumor-specific CD8+ T cell sequestration, dysfunction and deletion. Nat. Med..

[43] Khong H., Overwijk W.W. (2016). Adjuvants for peptide-based cancer vaccines. J. Immunother. Cancer.

[44] Walter E., Dreher D., Kok M., Thiele L., Kiama S.G., Gehr P., Merkle H.P. (2001). Hydrophilic poly (DL-lactide-co-glycolide) microspheres for the delivery of DNA to human-derived macrophages and dendritic cells. J. Control. Release.

[45] Serda R.E. (2013). Particle platforms for cancer immunotherapy. Int. J. Nanomed..

[46] Chittasupho C., Lirdprapamongkol K., Kewsuwan P., Sarisuta N. (2014). Targeted delivery of doxorubicin to A549 lung cancer cells by CXCR4 antagonist conjugated PLGA nanoparticles. Eur. J. Pharm. Biopharm..

[47] Guan Y.-Y., Luan X., Xu J.-R., Liu Y.-R., Lu Q., Wang C., Liu H.-J., Gao Y.-G., Chen H.-Z., Fang C. (2014). Selective eradication of tumor vascular pericytes by peptide-conjugated nanoparticles for antiangiogenic therapy of melanoma lung metastasis. Biomaterials.

[48] Wang X., Yang C., Zhang Y., Zhen X., Wu W., Jiang X. (2014). Delivery of platinum (IV) drug to subcutaneous tumor and lung metastasis using bradykinin-potentiating peptide-decorated chitosan nanoparticles. Biomaterials.

[49] Xiao Y.-F., Jie M.-M., Li B.-S., Hu C.-J., Xie R., Tang B., Yang S.-M. (2015). Peptide-based treatment: A promising cancer therapy. J. Immunol. Res..

[50] Bosteels V., Maréchal S., De Nolf C., Rennen S., Maelfait J., Tavernier S.J., Vetters J., Van De Velde E., Fayazpour F., Deswarte K. (2023). LXR signaling controls homeostatic dendritic cell maturation. Sci. Immunol..

[51] Prasad V., Kaestner V. (2017). Nivolumab and pembrolizumab: Monoclonal antibodies against programmed cell death-1 (PD-1) that are interchangeable. Proc. Semin. Oncol..

[52] Lee H.T., Lee J.Y., Lim H., Lee S.H., Moon Y.J., Pyo H.J., Ryu S.E., Shin W., Heo Y.-S. (2017). Molecular mechanism of PD-1/PD-L1 blockade via anti-PD-L1 antibodies atezolizumab and durvalumab. Sci. Rep..

[53] Lipson E.J., Drake C.G. (2011). Ipilimumab: An Anti-CTLA-4 Antibody for Metastatic MelanomaIpilimumab for Metastatic Melanoma. Clin. Cancer Res..

[54] Harding J.J., Moreno V., Bang Y.-J., Hong M.H., Patnaik A., Trigo J., Szpurka A.M., Yamamoto N., Doi T., Fu S. (2021). Blocking TIM-3 in treatment-refractory advanced solid tumors: A phase Ia/b study of LY3321367 with or without an anti-PD-L1 antibody. Clin. Cancer Res..

[55] Rousseau A., Parisi C., Barlesi F. (2023). Anti-TIGIT therapies for solid tumors: A systematic review. ESMO Open.

[56] Tagliamento M., Bironzo P., Novello S. (2020). New emerging targets in cancer immunotherapy: The role of VISTA. ESMO Open.

[57] Reck M., Rodríguez-Abreu D., Robinson A.G., Hui R., Csőszi T., Fülöp A., Gottfried M., Peled N., Tafreshi A., Cuffe S. (2016). Pembrolizumab versus chemotherapy for PD-L1–positive non–small-cell lung cancer. N. Engl. J. Med..

[58] Hellmann M.D., Ciuleanu T.-E., Pluzanski A., Lee J.S., Otterson G.A., Audigier-Valette C., Minenza E., Linardou H., Burgers S., Salman P. (2018). Nivolumab plus ipilimumab in lung cancer with a high tumor mutational burden. N. Engl. J. Med..

[59] Eng C., Kim T.W., Bendell J., Argilés G., Tebbutt N.C., Di Bartolomeo M., Falcone A., Fakih M., Kozloff M., Segal N.H. (2019). Atezolizumab with or without cobimetinib versus regorafenib in previously treated metastatic colorectal cancer (IMblaze370): A multicentre, open-label, phase 3, randomised, controlled trial. Lancet Oncol..

[60] Hung A.L., Maxwell R., Theodros D., Belcaid Z., Mathios D., Luksik A.S., Kim E., Wu A., Xia Y., Garzon-Muvdi T. (2018). TIGIT and PD-1 dual checkpoint blockade enhances antitumor immunity and survival in GBM. Oncoimmunology.

[61] Qin S., Xu L., Yi M., Yu S., Wu K., Luo S. (2019). Novel immune checkpoint targets: Moving beyond PD-1 and CTLA-4. Mol. Cancer.

[62] Lahiri A., Maji A., Potdar P.D., Singh N., Parikh P., Bisht B., Mukherjee A., Paul M.K. (2023). Lung cancer immunotherapy: Progress, pitfalls, and promises. Mol. Cancer.

[63] Ding Z., Li Q., Zhang R., Xie L., Shu Y., Gao S., Wang P., Su X., Qin Y., Wang Y. (2021). Personalized neoantigen pulsed dendritic cell vaccine for advanced lung cancer. Signal Transduct. Target. Ther..

[64] First Line IRESSA™ Versus Carboplatin/Paclitaxel in Asia. https://clinicaltrials.gov/study/NCT00322452.

[65] CCL21-Gene Modified Dendritic Cell Vaccine and Pembrolizumab in Treating Patients With Stage IV Non-small Cell Lung Cancer. https://clinicaltrials.gov/study/NCT03546361.

[66] Personalized DC Vaccines in Non Small Cell Lung Cancer. https://clinicaltrials.gov/study/NCT05195619.

[67] Wu Y.L., Chu D.T., Han B., Liu X., Zhang L., Zhou C., Liao M., Mok T., Jiang H., Duffield E. (2012). Phase III, randomized, open-label, first-line study in Asia of gefitinib versus carboplatin/paclitaxel in clinically selected patients with advanced non-small-cell lung cancer: Evaluation of patients recruited from mainland China. Asia Pac. J. Clin. Oncol..

[68] Personalized DC Vaccine for Postoperative Cancer. https://clinicaltrials.gov/study/NCT04147078.

[69] Study of Autologous CIK Cell Immunotherapy Combination With PD-1 Inhibitor and Chemotherapy in the Advanced NSCLC. https://clinicaltrials.gov/study/NCT03987867.

[70] Anti-PD-1 Alone or Combined With Autologous Cell Therapy in Advanced NSCLC. https://clinicaltrials.gov/study/NCT03360630.

[71] CIK Cell Transfusion Plus Gefitinib As Second Or Third-Line Treatment for Advanced Adenocarcinoma Non-Small Cell Lung Cancer. https://clinicaltrials.gov/study/NCT01871480.

[72] MK2206 and Erlotinib Hydrochloride in Treating Patients With Advanced Non-Small Cell Lung Cancer Who Have Progressed After Previous Response to Erlotinib Hydrochloride Therapy. https://clinicaltrials.gov/study/NCT01294306.

[73] Safety and Efficacy of DC-CIK in Patients With Advanced Non-Small-Cell Lung Cancer With Bone Metastases. https://clinicaltrials.gov/study/NCT02688686.

[74] Neoantigen-primed DC Vaccines Therapy for Refractory Lung Cancer. https://clinicaltrials.gov/study/NCT03871205.

[75] Combination Immunotherapy-Ipilimumab-Nivolumab-Dendritic Cell p53 Vac-Patients With Small Cell Lung Cancer (SCLC). https://clinicaltrials.gov/study/NCT03406715.

[76] Personalized DC Vaccine for Lung Cancer. https://clinicaltrials.gov/study/NCT02956551.

[77] Frankiw L., Baltimore D., Li G. (2019). Alternative mRNA splicing in cancer immunotherapy. Nat. Rev. Immunol..

[78] Xie N., Shen G., Gao W., Huang Z., Huang C., Fu L. (2023). Neoantigens: Promising targets for cancer therapy. Signal Transduct. Target. Ther..

[79] Rosenthal R., Cadieux E.L., Salgado R., Bakir M.A., Moore D.A., Hiley C.T., Lund T., Tanić M., Reading J.L., Joshi K. (2019). Neoantigen-directed immune escape in lung cancer evolution. Nature.

[80] Jaeger A.M., Stopfer L., Lee S., Gaglia G., Sandel D., Santagata S., Lin N.U., Trepel J.B., White F., Jacks T. (2019). Rebalancing protein homeostasis enhances tumor antigen presentation. Clin. Cancer Res..

[81] Anagnostou V., Smith K.N., Forde P.M., Niknafs N., Bhattacharya R., White J., Zhang T., Adleff V., Phallen J., Wali N. (2017). Evolution of neoantigen landscape during immune checkpoint blockade in non–small cell lung cancerdynamics of neoantigen landscape during immunotherapy. Cancer Discov..

[82] Liu Y., Xie C., Zhai Z., Deng Z.-y., De Jonge H.R., Wu X., Ruan Z. (2021). Uridine attenuates obesity, ameliorates hepatic lipid accumulation and modifies the gut microbiota composition in mice fed with a high-fat diet. Food Funct..

[83] Kreiter S., Vormehr M., Van de Roemer N., Diken M., Löwer M., Diekmann J., Boegel S., Schrörs B., Vascotto F., Castle J.C. (2015). Mutant MHC class II epitopes drive therapeutic immune responses to cancer. Nature.

[84] Lee M.Y., Jeon J.W., Sievers C., Allen C.T. (2020). Antigen processing and presentation in cancer immunotherapy. J. Immunother. Cancer.

[85] DePeaux K., Delgoffe G.M. (2021). Metabolic barriers to cancer immunotherapy. Nat. Rev. Immunol..

[86] Rizvi N.A., Hellmann M.D., Snyder A., Kvistborg P., Makarov V., Havel J.J., Lee W., Yuan J., Wong P., Ho T.S. (2015). Mutational landscape determines sensitivity to PD-1 blockade in non–small cell lung cancer. Science.

[87] Lawrence M.S., Stojanov P., Polak P., Kryukov G.V., Cibulskis K., Sivachenko A., Carter S.L., Stewart C., Mermel C.H., Roberts S.A. (2013). Mutational heterogeneity in cancer and the search for new cancer-associated genes. Nature.

[88] Hao J.-J., Lin D.-C., Dinh H.Q., Mayakonda A., Jiang Y.-Y., Chang C., Jiang Y., Lu C.-C., Shi Z.-Z., Xu X. (2016). Spatial intratumoral heterogeneity and temporal clonal evolution in esophageal squamous cell carcinoma. Nat. Genet..

[89] Levine A.J., Jenkins N.A., Copeland N.G. (2019). The roles of initiating truncal mutations in human cancers: The order of mutations and tumor cell type matters. Cancer Cell.

[90] Li F., Deng L., Jackson K.R., Talukder A.H., Katailiha A.S., Bradley S.D., Zou Q., Chen C., Huo C., Chiu Y. (2021). Neoantigen vaccination induces clinical and immunologic responses in non-small cell lung cancer patients harboring EGFR mutations. J. Immunother. Cancer.

[91] Lennerz V., Fatho M., Gentilini C., Frye R.A., Lifke A., Ferel D., Wölfel C., Huber C., Wölfel T. (2005). The response of autologous T cells to a human melanoma is dominated by mutated neoantigens. Proc. Natl. Acad. Sci. USA.

[92] Mandelboim O., Berke G., Fridkin M., Feldman M., Eisenstein M., Eisenbach L. (1994). CTL induction by a tumour-associated antigen octapeptide derived from a murine lung carcinoma. Nature.

[93] Mandelboim O., Vadai E., Fridkin M., Katz-Hillel A., Feldman M., Berke G., Eisenbach L. (1995). Regression of established murine carcinoma metastases following vaccination with tumour-associated antigen peptides. Nat. Med..

[94] Sensi M., Anichini A. (2006). Unique tumor antigens: Evidence for immune control of genome integrity and immunogenic targets for T cell-mediated patient-specific immunotherapy. Clin. Cancer Res..

[95] Blass E., Ott P.A. (2021). Advances in the development of personalized neoantigen-based therapeutic cancer vaccines. Nat. Rev. Clin. Oncol..

[96] Dagogo-Jack I., Shaw A.T. (2018). Tumour heterogeneity and resistance to cancer therapies. Nat. Rev. Clin. Oncol..

[97] Hanahan D., Weinberg R.A. (2011). Hallmarks of cancer: The next generation. Cell.

[98] Greaves M., Maley C.C. (2012). Clonal evolution in cancer. Nature.

[99] Hu Z., Ott P.A., Wu C.J. (2018). Towards personalized, tumour-specific, therapeutic vaccines for cancer. Nat. Rev. Immunol..

[100] George S., Miao D., Demetri G.D., Adeegbe D., Rodig S.J., Shukla S., Lipschitz M., Amin-Mansour A., Raut C.P., Carter S.L. (2017). Loss of PTEN Is Associated with Resistance to Anti-PD-1 Checkpoint Blockade Therapy in Metastatic Uterine Leiomyosarcoma. Immunity.

[101] Verdegaal E.M., de Miranda N.F., Visser M., Harryvan T., van Buuren M.M., Andersen R.S., Hadrup S.R., van der Minne C.E., Schotte R., Spits H. (2016). Neoantigen landscape dynamics during human melanoma-T cell interactions. Nature.

[102] Gerlinger M., Rowan A.J., Horswell S., Math M., Larkin J., Endesfelder D., Gronroos E., Martinez P., Matthews N., Stewart A. (2012). Intratumor heterogeneity and branched evolution revealed by multiregion sequencing. N. Engl. J. Med..

[103] Hilf N., Kuttruff-Coqui S., Frenzel K., Bukur V., Stevanović S., Gouttefangeas C., Platten M., Tabatabai G., Dutoit V., van der Burg S.H. (2019). Actively personalized vaccination trial for newly diagnosed glioblastoma. Nature.

[104] Keskin D.B., Anandappa A.J., Sun J., Tirosh I., Mathewson N.D., Li S., Oliveira G., Giobbie-Hurder A., Felt K., Gjini E. (2019). Neoantigen vaccine generates intratumoral T cell responses in phase Ib glioblastoma trial. Nature.

[105] Shen Y., Yu L., Xu X., Yu S., Yu Z. (2022). Neoantigen vaccine and neoantigen-specific cell adoptive transfer therapy in solid tumors: Challenges and future directions. Cancer Innov..

[106] Fritsch E.F., Rajasagi M., Ott P.A., Brusic V., Hacohen N., Wu C.J. (2014). HLA-Binding Properties of Tumor Neoepitopes in HumansTumor Neoepitopes in Humans. Cancer Immunol. Res..

[107] Peters B., Nielsen M., Sette A. (2020). T cell epitope predictions. Annu. Rev. Immunol..

[108] Sarkizova S., Klaeger S., Le P.M., Li L.W., Oliveira G., Keshishian H., Hartigan C.R., Zhang W., Braun D.A., Ligon K.L. (2020). A large peptidome dataset improves HLA class I epitope prediction across most of the human population. Nat. Biotechnol..

[109] Capietto A.-H., Jhunjhunwala S., Pollock S.B., Lupardus P., Wong J., Hänsch L., Cevallos J., Chestnut Y., Fernandez A., Lounsbury N. (2020). Mutation position is an important determinant for predicting cancer neoantigens. J. Exp. Med..

[110] Harari A., Graciotti M., Bassani-Sternberg M., Kandalaft L.E. (2020). Antitumour dendritic cell vaccination in a priming and boosting approach. Nat. Rev. Drug Discov..

[111] Siegel R.L., Miller K.D., Jemal A. (2019). Cancer statistics, 2019. CA Cancer J. Clin..

[112] Brunsvig P.F., Kyte J.A., Kersten C., Sundstrøm S., Møller M., Nyakas M., Hansen G.L., Gaudernack G., Aamdal S. (2011). Telomerase Peptide Vaccination in NSCLC: A Phase II Trial in Stage III Patients Vaccinated after Chemoradiotherapy and an 8-Year Update on a Phase I/II TrialTelomerase Peptide Vaccination in NSCLC. Clin. Cancer Res..

[113] Butts C., Socinski M.A., Mitchell P.L., Thatcher N., Havel L., Krzakowski M., Nawrocki S., Ciuleanu T.-E., Bosquée L., Trigo J.M. (2014). Tecemotide (L-BLP25) versus placebo after chemoradiotherapy for stage III non-small-cell lung cancer (START): A randomised, double-blind, phase 3 trial. Lancet Oncol..

[114] Nemunaitis J., Jahan T., Ross H., Sterman D., Richards D., Fox B., Jablons D., Aimi J., Lin A., Hege K. (2006). Phase 1/2 trial of autologous tumor mixed with an allogeneic GVAX^®^ vaccine in advanced-stage non-small-cell lung cancer. Cancer Gene Ther..

[115] Vansteenkiste J.F., Cho B.C., Vanakesa T., De Pas T., Zielinski M., Kim M.S., Jassem J., Yoshimura M., Dahabreh J., Nakayama H. (2016). Efficacy of the MAGE-A3 cancer immunotherapeutic as adjuvant therapy in patients with resected MAGE-A3-positive non-small-cell lung cancer (MAGRIT): A randomised, double-blind, placebo-controlled, phase 3 trial. Lancet Oncol..

[116] Thomas A., Giaccone G. (2015). Why has active immunotherapy not worked in lung cancer?. Ann. Oncol..

[117] Kalinski P., Urban J., Narang R., Berk E., Wieckowski E., Muthuswamy R. (2009). Dendritic cell-based therapeutic cancer vaccines: What we have and what we need. Future Oncol..

[118] Steinman R.M., Cohn Z.A. (1973). Identification of a novel cell type in peripheral lymphoid organs of mice: I. Morphology, quantitation, tissue distribution. J. Exp. Med..

[119] Steinman R.M., Banchereau J. (2007). Taking dendritic cells into medicine. Nature.

[120] Del Prete A., Salvi V., Soriani A., Laffranchi M., Sozio F., Bosisio D., Sozzani S. (2023). Dendritic cell subsets in cancer immunity and tumor antigen sensing. Cell. Mol. Immunol..

[121] Duong E., Fessenden T.B., Lutz E., Dinter T., Yim L., Blatt S., Bhutkar A., Wittrup K.D., Spranger S. (2022). Type I interferon activates MHC class I-dressed CD11b+ conventional dendritic cells to promote protective anti-tumor CD8+ T cell immunity. Immunity.

[122] Bloemendal M., Bol K.F., Boudewijns S., Gorris M.A., de Wilt J.H., Croockewit S.A., van Rossum M.M., de Goede A.L., Petry K., Koornstra R.H. (2022). Immunological responses to adjuvant vaccination with combined CD1c+ myeloid and plasmacytoid dendritic cells in stage III melanoma patients. Oncoimmunology.

[123] Patente T.A., Pinho M.P., Oliveira A.A., Evangelista G.C., Bergami-Santos P.C., Barbuto J.A. (2019). Human dendritic cells: Their heterogeneity and clinical application potential in cancer immunotherapy. Front. Immunol..

[124] Dudek A.M., Martin S., Garg A.D., Agostinis P. (2013). Immature, semi-mature, and fully mature dendritic cells: Toward a DC-cancer cells interface that augments anticancer immunity. Front. Immunol..

[125] Fucikova J., Palova-Jelinkova L., Bartunkova J., Spisek R. (2019). Induction of tolerance and immunity by dendritic cells: Mechanisms and clinical applications. Front. Immunol..

[126] Murgaski A., Kiss M., Van Damme H., Kancheva D., Vanmeerbeek I., Keirsse J., Hadadi E., Brughmans J., Arnouk S.M., Hamouda A.E. (2022). Efficacy of CD40 agonists is mediated by distinct cDC subsets and subverted by suppressive macrophages. Cancer Res..

[127] Fong L., Hou Y., Rivas A., Benike C., Yuen A., Fisher G.A., Davis M.M., Engleman E.G. (2001). Altered peptide ligand vaccination with Flt3 ligand expanded dendritic cells for tumor immunotherapy. Proc. Natl. Acad. Sci. USA.

[128] Itoh T., Ueda Y., Kawashima I., Nukaya I., Fujiwara H., Fuji N., Yamashita T., Yoshimura T., Okugawa K., Iwasaki T. (2002). Immunotherapy of solid cancer using dendritic cells pulsed with the HLA-A24-restricted peptide of carcinoembryonic antigen. Cancer Immunol. Immunother..

[129] Ueda Y., Itoh T., Nukaya I., Kawashima I., Okugawa K., Yano Y., Yamamoto Y., Naitoh K., Shimizu K., Imura K. (2004). Dendritic cell-based immunotherapy of cancer with carcinoembryonic antigen-derived, HLA-A24-restricted CTL epitope: Clinical outcomes of 18 patients with metastatic gastrointestinal or lung adenocarcinomas. Int. J. Oncol..

[130] Quoix E., Lena H., Losonczy G., Forget F., Chouaid C., Papai Z., Gervais R., Ottensmeier C., Szczesna A., Kazarnowicz A. (2016). TG4010 immunotherapy and first-line chemotherapy for advanced non-small-cell lung cancer (TIME): Results from the phase 2b part of a randomised, double-blind, placebo-controlled, phase 2b/3 trial. Lancet Oncol..

[131] Hirschowitz E.A., Foody T., Hidalgo G.E., Yannelli J.R. (2007). Immunization of NSCLC patients with antigen-pulsed immature autologous dendritic cells. Lung Cancer.

[132] Wang S., Wang Z. (2015). Efficacy and safety of dendritic cells co-cultured with cytokine-induced killer cells immunotherapy for non-small-cell lung cancer. Int. Immunopharmacol..

[133] Gao X., Mi Y., Guo N., Xu H., Xu L., Gou X., Jin W. (2017). Cytokine-induced killer cells as pharmacological tools for cancer immunotherapy. Front. Immunol..

[134] Yang L., Ren B., Li H., Yu J., Cao S., Hao X., Ren X. (2013). Enhanced antitumor effects of DC-activated CIKs to chemotherapy treatment in a single cohort of advanced non-small-cell lung cancer patients. Cancer Immunol. Immunother..

[135] Zhao P., Bu X., Wei X., Sun W., Xie X., Li C., Guo Q., Zhu D., Wei X., Gao D. (2015). Dendritic cell immunotherapy combined with cytokine-induced killer cells promotes skewing toward Th2 cytokine profile in patients with metastatic non-small cell lung cancer. Int. Immunopharmacol..

[136] Zhang L., Yang X., Sun Z., Li J., Zhu H., Li J., Pang Y. (2016). Dendritic cell vaccine and cytokine-induced killer cell therapy for the treatment of advanced non-small cell lung cancer. Oncol. Lett..

[137] Song H., Liu S., Zhao Z., Sun W., Wei X., Ma X., Zhao P., Gao D. (2017). Increased cycles of DC/CIK immunotherapy decreases frequency of Tregs in patients with resected NSCLC. Int. Immunopharmacol..

[138] Zhao Y., Qiao G., Wang X., Song Y., Zhou X., Jiang N., Zhou L., Huang H., Zhao J., Morse M. (2019). Combination of DC/CIK adoptive T cell immunotherapy with chemotherapy in advanced non-small-cell lung cancer (NSCLC) patients: A prospective patients’ preference-based study (PPPS). Clin. Transl. Oncol..

[139] Zhu X., Xu Y., Zhou J., Pan X. (2015). A clinical study evaluating dendritic and cytokine-induced killer cells combined with concurrent radiochemotherapy for stage IIIB non-small cell lung cancer. Genet. Mol. Res..

[140] Kimura H., Iizasa T., Ishikawa A., Shingyouji M., Yoshino M., Kimura M., Inada Y., Matsubayashi K. (2008). Prospective phase II study of post-surgical adjuvant chemo-immunotherapy using autologous dendritic cells and activated killer cells from tissue culture of tumor-draining lymph nodes in primary lung cancer patients. Anticancer Res..

[141] Kimura H., Matsui Y., Ishikawa A., Nakajima T., Iizasa T. (2018). Randomized controlled phase III trial of adjuvant chemoimmunotherapy with activated cytotoxic T cells and dendritic cells from regional lymph nodes of patients with lung cancer. Cancer Immunol. Immunother..

[142] Antonia S.J., López-Martin J.A., Bendell J., Ott P.A., Taylor M., Eder J.P., Jäger D., Pietanza M.C., Le D.T., de Braud F. (2016). Nivolumab alone and nivolumab plus ipilimumab in recurrent small-cell lung cancer (CheckMate 032): A multicentre, open-label, phase 1/2 trial. Lancet Oncol..

[143] Chung H.C., Piha-Paul S.A., Lopez-Martin J., Schellens J.H., Kao S., Miller Jr W.H., Delord J.-P., Gao B., Planchard D., Gottfried M. (2020). Pembrolizumab after two or more lines of previous therapy in patients with recurrent or metastatic SCLC: Results from the KEYNOTE-028 and KEYNOTE-158 studies. J. Thorac. Oncol..

[144] Chiappori A.A., Soliman H., Janssen W.E., Antonia S.J., Gabrilovich D.I. (2010). INGN-225: A dendritic cell-based p53 vaccine (Ad. p53-DC) in small cell lung cancer: Observed association between immune response and enhanced chemotherapy effect. Expert Opin. Biol. Ther..

[145] Antonia S.J., Mirza N., Fricke I., Chiappori A., Thompson P., Williams N., Bepler G., Simon G., Janssen W., Lee J.-H. (2006). Combination of p53 cancer vaccine with chemotherapy in patients with extensive stage small cell lung cancer. Clin. Cancer Res..

[146] Chiappori A.A., Williams C.C., Gray J.E., Tanvetyanon T., Haura E.B., Creelan B.C., Thapa R., Chen D.-T., Simon G.R., Bepler G. (2019). Randomized-controlled phase II trial of salvage chemotherapy after immunization with a TP53-transfected dendritic cell-based vaccine (Ad. p53-DC) in patients with recurrent small cell lung cancer. Cancer Immunol. Immunother..

[147] Iclozan C., Antonia S., Chiappori A., Chen D.-T., Gabrilovich D. (2013). Therapeutic regulation of myeloid-derived suppressor cells and immune response to cancer vaccine in patients with extensive stage small cell lung cancer. Cancer Immunol. Immunother..

[148] Stevens D., Ingels J., Van Lint S., Vandekerckhove B., Vermaelen K. (2021). Dendritic cell-based immunotherapy in lung cancer. Front. Immunol..

[149] Verdijk P., Aarntzen E.H., Lesterhuis W.J., Boullart A.I., Kok E., van Rossum M.M., Strijk S., Eijckeler F., Bonenkamp J.J., Jacobs J.F. (2009). Limited amounts of dendritic cells migrate into the T-cell area of lymph nodes but have high immune activating potential in melanoma patients. Clin. Cancer Res..

